# Evaluation of the immunotoxicity potential of nanomaterials using THP-1 cells

**DOI:** 10.3389/ftox.2024.1293147

**Published:** 2024-07-01

**Authors:** Asuka Nishida, Yuka Sawada, Rion Arai, Naoki Ishibashi, Miho Suzuo, Akiko Ohno, Takao Ashikaga, Kazutoshi Iijima

**Affiliations:** ^1^ Graduate School of Engineering Science, Yokohama National University, Yokohama, Japan; ^2^ College of Engineering Science, Yokohama National University, Yokohama, Japan; ^3^ Division of Risk Assessment, National Institute of Health Sciences, Kawasaki, Japan; ^4^ Faculty of Engineering, Yokohama National University, Yokohama, Japan

**Keywords:** nanomaterials, immunotoxicity, THP-1 cells, antigen-presenting cells, silver, titanium dioxide, silica

## Abstract

With the expansion of nanomaterials (NMs) usage, concerns about their toxicity are increasing, and the wide variety of NMs makes it difficult to assess their toxicity. Therefore, the development of a high-throughput, accurate, and certified method to evaluate the immunotoxicity of NMs is required. In this study, we assessed the immunotoxicity potential of various NMs, such as nanoparticles of silver, silica, and titanium dioxide, using the human Cell Line Activation Test (h-CLAT) at the cellular level. After exposure to silver nanoparticle dispersions, the expression levels of CD86 and CD54 increased, suggesting the activation of antigen-presenting cells (APCs) by silver nanoparticles. Quantification of silver ions eluted from silver nanoparticles and the activation of APCs by silver ions suggested that it was due to the release of silver ions. Silica nanoparticles also increased the expression of CD86 and/or CD54, and their activation ability correlated with the synthesis methods and hydrodynamic diameters. The ability of titanium dioxide to activate APCs differed depending on the crystal type and hydrodynamic diameter. These results suggest a potential method to evaluate the immunotoxicity potential of various NMs based on their ability to activate APCs using human monocytic THP-1 cells. This method will be valuable in assessing the immunotoxicity potential and elucidating the immunotoxic mechanisms of NMs.

## 1 Introduction

Nanomaterials (NMs) possess unique properties that are not found in the bulk state and have been utilized in a wide range of industries, including electronics (Wu, 2017), energy (Wang et al., 2020), medicine (Sim and Wong, 2021; Dadhwal et al., 2023), food (McClements and Xiao, 2017), and cosmetics (Fytianos et al., 2020). With the increasing use of NMs, their toxicity concerns are growing. NMs can be absorbed into the body through the respiratory, percutaneous, and digestive organs and show toxicity mainly through three mechanisms: direct association with the cell membrane, dissolution to release toxic ions, and oxidative stress (Buchman et al., 2019). One of the difficult factors in assessing the toxicity of NMs is their wide variety. For example, NMs containing the same substance as the main component have shown different toxicities depending on the particle size, shape, specific surface area, crystal type, impurities, and surface coating (Sharifi et al., 2012). Therefore, it is challenging to construct a database for predicting the toxicity of NMs like small-molecular compounds. Furthermore, establishment of a method for evaluating the toxicity of NMs will lead to the development of metal NMs with mitigated toxicity (Zhang et al., 2022).

NMs toxicity has been evaluated mainly in laboratory animals. Inhalation exposure of multi-walled carbon nanotubes in mice promoted chronic inflammation and formation of fibrotic lesion in macrophages (Otsuka et al., 2018). In rats, a pulmonary inflammatory response was induced by amorphous silica nanoparticles (NPs) and the effects depended on the particle size (Han et al., 2020). Intratracheal exposure of titanium dioxide (TiO_2_) NMs to mice induced pulmonary inflammation and alveolar proteinosis (Danielsen et al., 2020). However, in the past decade, *in vitro* models have been developed to more easily evaluate the toxicity of NMs and establish an adverse outcome pathway (AOP) of NMs (Tirumala et al., 2021).

To date, the immunotoxicity of NMs has been independently investigated using several kinds of cells, such as murine dendritic cells (Winter et al., 2011), murine macrophage-like RAW 264.7 cells (Alsaleh et al., 2019), rat NR8383 alveolar macrophages (Wiemann et al., 2016), murine macrophage cell line J774A.1 cells and human lung adenocarcinoma cell line A549 cells (Breznan et al., 2017), human monocytic cell line THP-1 cells (Nishijima et al., 2017a; Cui et al., 2020; Murugadoss et al., 2020; Eto et al., 2022), and differentiated THP-1 cells (Brzicova et al., 2019) with a variety of evaluation indices, such as CD80, CD86, CD54, major histocompatibility complex (MHC)-II, interleukin (IL)-1β, IL-6, and tumor necrosis factor (TNF)-α. Therefore, it is essential to develop a standardized *in vitro* method to evaluate the immunotoxicity of NMs.

In this study, we assessed the immunotoxicity potential of NMs using the human Cell Line Activation Test (h-CLAT) (Ashikaga et al., 2006) at the cellular level, with the aim of developing an *in vitro* method for NMs immunotoxicity. High-throughput and reliable *in vitro* test, corresponding to an antigen presenting cell activation, should be a useful method as a screening for NMs immunotoxicity. h-CLAT is a validated standard method for evaluating the skin sensitization potential of chemicals listed in the Organization for Economic Co-operation and Development (OECD) test guidelines (OECD, 2018). In h-CLAT, post-exposure to test substances, the expression levels of CD86 and CD54 (surface antigens of the human monocytic cell line THP-1) are used as an indicator of dendritic cell activation (OECD, 2018). The novelty of this study is that the degree of activation of THP-1 cells exposed to various nanomaterials were quantified with h-CLAT and compared among the samples with different chemical composition and physical properties.

## 2 Materials and methods

### 2.1 Reagents

Silver NPs (BioPureTM Silver Nanospheres, AGCB10, and AGCB50) were purchased from nanoComposix (San Diego, CA, United States of America). Synthetic amorphous silica NPs (NM-200 (JRCNM02000a990281), NM-201 (JRCNM02001a990208), NM-202 (JRCNM02002a990589), NM-203 (JRCNM10404a020037), and NM-204 (JRCNM02004a990075)) were kindly provided from the European Commission’s Joint Research Centre (JRC) Nanomaterial Repository. The silica NP (Sicastar-red F) was purchased from Micromod Partikeltechnologie GmbH (Rostock, Germany). The TiO_2_ NPs (MT-150A, MT-500B, AMT-100, AMT-600, and TKP-102) were obtained from Tayca Corp. (Osaka, Japan). All silica NPs and TiO_2_ NPs were dry heated at 220°C for 18 h in a dry heat sterilizer (STA620DA, Advantec Toyo Kaisha, Ltd.) or muffle furnace (1-6033-12, AS ONE Corp., Osaka, Japan) to eliminate the effect of contaminated lipopolysaccharides. All other reagents and chemicals were used as received. Ultrapure water prepared in Direct-Q UV5 (Merck Millipore, Burlington, MA, United States of America) or Milli-Q Integral 10 (Merck Millipore) was used for all experiments.

### 2.2 Cell culture

The human monocytic leukemia cell line, THP-1, was purchased from the American Type Culture Collection (Manassas, VA, United States of America) and maintained in RPMI-1640 medium containing l-glutamate supplemented with 10% (v/v) heat-inactivated fetal bovine serum (FBS, Corning Inc., Corning, NY, United States of America), 0.1% (v/v) 2-mercaptoethanol (Thermo Fisher Scientific), and 1% (v/v) penicillin-streptomycin solution (Nacalai Tesque, Kyoto, Japan) (RPMI-1640 (+)) at 37°C and 5% CO_2_.

### 2.3 Preparation of NM dispersions

Silver NP dispersions were prepared according to a previous report (Hirai et al., 2016). Briefly, the purchased silver NPs dispersions (1 mg/mL) were re-dispersed using HI-TECH MIXER (M-90001, Hi-Tech Co., Ltd., Tokyo, Japan) for 10 s and mixed with a 40 mg/mL albumin from Bovine serum (BSA, Fujifilm Wako Pure Chemical Corp., Osaka, Japan) solution in 5% glucose solution at a ratio of 1:1 (v/v). The concentrations required for each experiment were prepared in the RPMI-1640 (+). Silica NPs (NM-200, NM-201, NM-202, NM-203, and NM-204) were added to ultrapure water at a concentration of 20 mg/mL and sonicated on ice using a tip ultrasonicator (Digital Sonifer 250D, Branson Ultrasonics Corp., Brookfield, CT, United States of America) at 40 W for 5 min twice at 15 min interval. After dispersion, the solution was diluted with RPMI-1640 (+) to prepare the working solution. Similarly, Sicastar-red F was diluted in RPMI-1640 (+) and sonicated at 15 W for 20 s using a tip ultrasonicator to prepare the working solution. The TiO_2_ NPs (MT-150A, MT-500B, AMT-100, AMT-600, and TKP-102) were added to RPMI-1640 (+) at a concentration of 4 mg/mL and sonicated on ice using a tip ultrasonicator (VP-050N, Taitec Corp., Saitama, Japan) at 40 W for 1 min. After dispersion, the solution was diluted with RPMI-1640 (+) and sonicated at 40 W for 1 min to prepare a working solution. The dispersion states of the stock and working solutions are summarized in Supplementary Table S1.

### 2.4 Measurement of diameter and ζ-potential of NMs

The diameter and ζ-potential of NMs were measured in RPMI-1640 (+) using a ζ-potential & dynamic light scattering (DLS) analyzer (ELSZ-2, Otsuka Electronics Co., Ltd., Osaka, Japan). Each experiment was done in triplicate, and the results are shown as mean ± standard deviation (S.D.).

### 2.5 Inductively coupled plasma atomic emission spectroscopy (ICP-AES)

The purchased silver NP dispersions were re-dispersed using HI-TECH MIXER for 10 s and diluted 2-fold with ultrapure water in a 1.5 mL tube. After centrifugation (3700, Kubota Corp., Tokyo, Japan) at 4°C and 21,880 ×g for 75 min, the supernatant was diluted 25-fold with ultrapure water and filtered using a 0.22 μm Millex filter (Merck KGaA, Darmstadt, Germany). The silver content was quantified using ICP-AES (ICPE-9000, Shimadzu Corp., Kyoto, Japan) at 328.068 nm with reference to a standard curve prepared using silver nitrate solution (0.08–15.7 μg/mL). The concentration of silver ions eluted under the cell culture conditions was also quantified using ICP-AES. The silver NP dispersions in RPMI-1640 (+) were at a concentration of CV75, the dose estimated to give 75% cell viability (201.2 and 242.2 μg/mL for AGCB10 and AGCB50, respectively), and incubated at 37°C for 24 h in a 5% CO_2_ incubator. After centrifugation (21,880 ×g, 75 min, 4°C), the silver content in the supernatant was quantified using a similar procedure described above.

### 2.6 Exposure of NMs to THP-1 cells and flow cytometric analysis

The expression levels of CD86 and CD54 cell surface markers and cell viability of THP-1 cells were analyzed according to the h-CLAT protocol (OECD, 2018) with slight modifications. Briefly, 1.0 × 10^6^ THP-1 cells in 500 μL RPMI-1640 (+) were seeded in each well of a 24-well plate, and 500 μL of NM dispersion was added and incubated at 37°C and 5% CO_2_ for 24 h. The cells were collected in sample tubes and washed twice with 1 mL of phosphate-buffered saline (PBS, pH 7.1–7.3) containing 0.1% (w/v) BSA (FACS buffer). The cells were then re-suspended with 600 μL of blocking solution containing 0.01% (w/v) globulins Cohn fractions II and III (Sigma-Aldrich Co. LLC, St. Louis MO, United States of America)) and divided into three aliquots into well of 96-well round plate. After incubation at 4°C for 15 min, plate was centrifuged at 2,500 rpm for 3 min using PlateSpin (Kubota) and the supernatant was removed. Pre-mixed antibody solutions (fluorescein isothiocyanate (FITC)-labeled anti-CD86 antibody (BD Pharmingen, San Diego, CA, United States of America), FITC-labeled anti-CD54 antibody (Dako, Glostrup, Denmark), or FITC-labeled mouse IgG1 (Dako) in FACS buffer were added to each cell pellet and incubated at 4°C for 30 min in the dark. The stained cells were washed twice with 200 μL of FACS buffer. The cells were re-suspended in 400 μL of FACS buffer and transferred to a 5 mL polystyrene round tube through a mesh (T-No380T, SANSYO Co., Ltd.). 10 μL of 25 μg/mL propidium iodide (PI) solution (Fujifilm Wako Pure Chemical Corp.) was added to measure cell viability. The expression level of CD86 and CD54 cell surface markers and cell viability of THP-1 cells were evaluated by flow cytometry using BD Accuri™ C6 Plus (BD Biosciences, Franklin Lakes, NJ, United States of America) or Navios EX (Beckman Coulter, Brea, CA, United States of America). Dead cells were gated out, and the mean fluorescence intensity (MFI) was acquired and the relative fluorescence intensity (RFI), which is an indicator of CD86 and CD54 expression were determined. Each experiment was done in triplicate, and the results are shown as the mean ± S.D. RFI was calculated using the following formula (1).

$$RFI=\frac{\left(MFI\;of\;test\;substance-treated\;cells\right)-\left(MFI\;of\;test\;substance-treated\;isotype\;control\;of\;cells\right)}{\left(MFI\;of\;solvent-treated\;control\;cells\right)-\left(\;MFI\;of\;solvent-treated\;isotype\;control\;cells\right)}*100$$ (1)

Nickel sulfate hexahydrate solution (final concentration: 100 μg/mL) was used as positive control in the measurement of silver NPs, AgNO_3_, Sicastar-red F, and TiO_2_ NPs. 2,4-Dinitrochlorobenzene (DNCB) solution (final concentration: 4 μg/mL) was used as positive control in the measurement of silica NPs excepting Sicastar-red F.

EC150 and EC200, the concentrations of NMs at which RFI (CD86) and RFI (CD54) are 150 and 200, respectively, and CV75, the concentration at which the cell viability is 75%, were calculated using formula (2) (OECD, 2018) and (3), respectively. The EC values could potentially contribute to the assessment of sensitizing potency (Jaworska et al., 2015). The smaller the values, the stronger the sensitization potency of the test substance.

$$\left.EC150\;or\;EC200=B_{concentration}+{\frac{150\left(CD86\right)or\;200\;\left(CD54\right)-B_{RFI}}{A_{RFI}-B_{RFI}}*(A}_{concentration}-B_{concentration}\right)$$ (2)

where A_concentration_ is the lowest concentration with RFI >150 (CD86) or 200 (CD54) B_concentration_ is the highest concentration with RFI <150 (CD86) or 200 (CD54).A_RFI_ is the RFI at A_concentration_B_RFI_ is the RFI at B_concentration_

$$\left.CV75=B_{concentration}+{\frac{75-B_{V}}{A_{V}-B_{V}}*(A}_{concentration}-B_{concentration}\right)$$ (3)

where A_concentration_ is the lowest concentration with cell viability >75.B_concentration_ is the highest concentration with cell viability <75.A_V_ is the cell viability at A_concentration_B_V_ is the cell viability at B_concentration_EC150, EC200 or CV75 were calculated from the data obtained in each experiment and the median of triplicate are shown.

## 3 Results and discussion

### 3.1 Characterization of NMs

The ζ-potential, hydrodynamic diameter, and polydispersity index (PDI) of NMs in RPMI-1640 (+) are summarized in Table 1 with the specifications described in the data sheets from manufacturers and the JRC Nanomaterial Repository [Table 1 near here].

**TABLE 1:** Properties of the nanomaterials used in this study.

	Name	Synthesis^a^	Crystal^a^	Shape	Size (TEM/SEM)^a^/nm	Size (DLS)^b^/nm	ζ-potential^b^/mV	PDI^b^/-
Ag	AGCB10			Particle	10 ± 2	38 ± 11	−12.1 ± 0.7	0.36 ± 0.03
	AGCB50			Particle	52 ± 6	72 ± 3	−17.0 ± 2.5	0.27 ± 0.02
SiO_2_	Sicastar-red F	Stöber	Amorphous	Particle	70	221 ± 48	−10.2 ± 2.1	0.34 ± 0.07
	NM-200	Precipitated	Amorphous	Particle	14 ± 7	331 ± 61	−21.0 ± 0.7	0.34 ± 0.02
	NM-201	Precipitated	Amorphous	Particle	17 ± 8	526 ± 70	−18.1 ± 1.3	0.38 ± 0.08
	NM-202	Thermal	Amorphous	Particle	15 ± 7	420 ± 124	−15.9 ± 0.2	0.36 ± 0.02
	NM-203	Thermal	Amorphous	Particle	13 ± 6	328 ± 38	−17.4 ± 1.1	0.42 ± 0.03
	NM-204	Precipitated	Amorphous	Particle	10–15	273 ± 105	−17.3 ± 0.8	0.35 ± 0.03
TiO_2_	MT-150A		Rutile	Particle	15	222 ± 21	−20.5 ± 3.3	0.22 ± 0.01
	MT-500B		Rutile	Particle	35	82 ± 30	−23.9 ± 4.0	0.23 ± 0.01
	AMT-100		Anatase	Particle	6	235 ± 60	−22.4 ± 0.8	0.14 ± 0.05
	AMT-600		Anatase	Particle	30	261 ± 24	−16.7 ± 3.9	0.21 ± 0.01
	TKP-102		Anatase	Particle	15	61 ± 12	−23.8 ± 1.3	0.27 ± 0.07

^a^ Datasheet from manufacturers and the European Commission Joint Research Centre (JRC).

^b^ Measured in RPMI-1640 (+) at CV75 concentration for Ag. Measured in RPMI-1640 (+) at 1,000 μg/mL concentration for SiO_2_ and TiO_2_.

PDI, polydispersity index; TEM, transmission electron microscopy; SEM, scanning electron microscopy.

### 3.2 Effect of silver NPs on CD86 and CD54 expression by THP-1 cells

**FIGURE 1:**
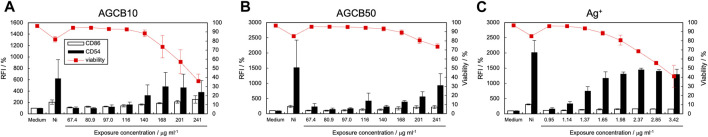
Effect of silver NPs on CD86 and CD54 expression. The expression of CD86 and CD54 and cell viability of THP-1 cells after exposure to silver NPs, AGCB10 (10 nm) **(A)** and AGCB50 (50 nm) **(B)**, and silver nitrate solution **(C)** were quantified using flow cytometry. Each experiment was performed in triplicate, and the results are shown as mean ± standard deviation (S.D.); RFI, relative fluorescent intensity. The concentration was expressed as the concentration of silver ions in **(C)**.

After exposure to silver NP dispersions of 10 nm (AGCB10, Figure 1A) and 50 nm (AGCB50, Figure 1B) and silver nitrate solution (Figure 1C), the expression levels of CD86 and CD54 increased and met the positive criteria of h-CLAT [Figure 1 near here]. In our previous study on the *in vitro* skin sensitization test: h-CLAT, the concentration at the chemical reaches positive criteria at certain period of time were correlated with the strength of sensitization potentials (Hirota et al., 2018). Therefore, the concentration at the chemical reaches positive criteria were calculated (Table 2). The EC150 and EC200 of silver ions were substantially lower than that of silver NPs [Table 2 near here]. These results suggest that the ability of silver ions to activate antigen-presenting cells (APCs) is significantly higher than that of silver NPs. Furthermore, the silver ion content in the AGCB10 and AGCB50 solutions 24 h after their preparation was 25.9% and 1.67%, respectively (Table 3). Silver ions are known as skin sensitizers (Hirai et al., 2016), indicating that the activation of APCs by silver NPs is due to the release of silver ions. In addition, EC150 of AGCB10 was lower than that of AGCB50. This result was consistent with a previous report in which inflammation induced in mice by exposure to silver NPs increased as the particle size decreased (Hirai et al., 2016). There are three possible mechanisms through which smaller-sized silver NPs can activate THP-1 cells. First is the difference in the cellular uptake of silver NPs depending on the particle size. It has been demonstrated that APCs, such as dendritic cells, preferentially take up smaller particles (Blank et al., 2013). Second is the difference in the concentration of eluted silver ions depending on the particle size. It was observed that more silver ions were eluted from silver NPs with smaller particle diameters (Table 3), and the higher concentrations of silver ions probably activated THP-1 cells [Table 3 near here]. Third is protein adsorbed on the surface of silver NPs. In this study, in order to improve the dispersity of silver NPs, these were mixed with albumin based on the reference (Hirai et al., 2016). Difference in albumin adsorption on the surface of silver particles may contribute to the difference of activation of THP-1 cells.

**TABLE 2:** CV75, EC150 (CD86), and EC200 (CD54) values of the nanomaterials.

	Name	CV75/μg mL^−1^	EC150 (CD86)/μg mL^−1^	EC200 (CD54)/μg mL^−1^
Ag	AGCB10	201.2	127.6	118.4
	AGCB50	242.2	159.5	122.9
	Ag^+^	2.37	1.64	0.98
SiO_2_	Sicastar-red F	286.8	-	1.11
	NM-200	-	-	10.3
	NM-201	-	-	30.3
	NM-202	821.7	-	19.5
	NM-203	857.7	-	24.8
	NM-204	-	-	3.48
TiO_2_	MT-150A	-	-	-
	MT-500B	-	-	-
	AMT-100	-	-	<7.81
	AMT-600	-	-	18.36
	TKP-102	-	223.2	16.29

(−) in CV75: >1,000 μg mL^−1^.

(−) in EC150 and EC200: “negative” at the tested concentrations.

CV75 for Sicastar-red F may be affected by the fluorescent modification of the particles.

**TABLE 3:** Quantification of silver ions released from silver NPs.

Name	% Of silver ions released from silver NPs
AGCB10	5.00
AGCB50	0.13
AGCB10 after 24 h in RPMI	25.9
AGCB50 after 24 h In RPMI	1.67

### 3.3 Effect of silica NPs on CD86 and CD54 expression by THP-1 cells

**FIGURE 2:**
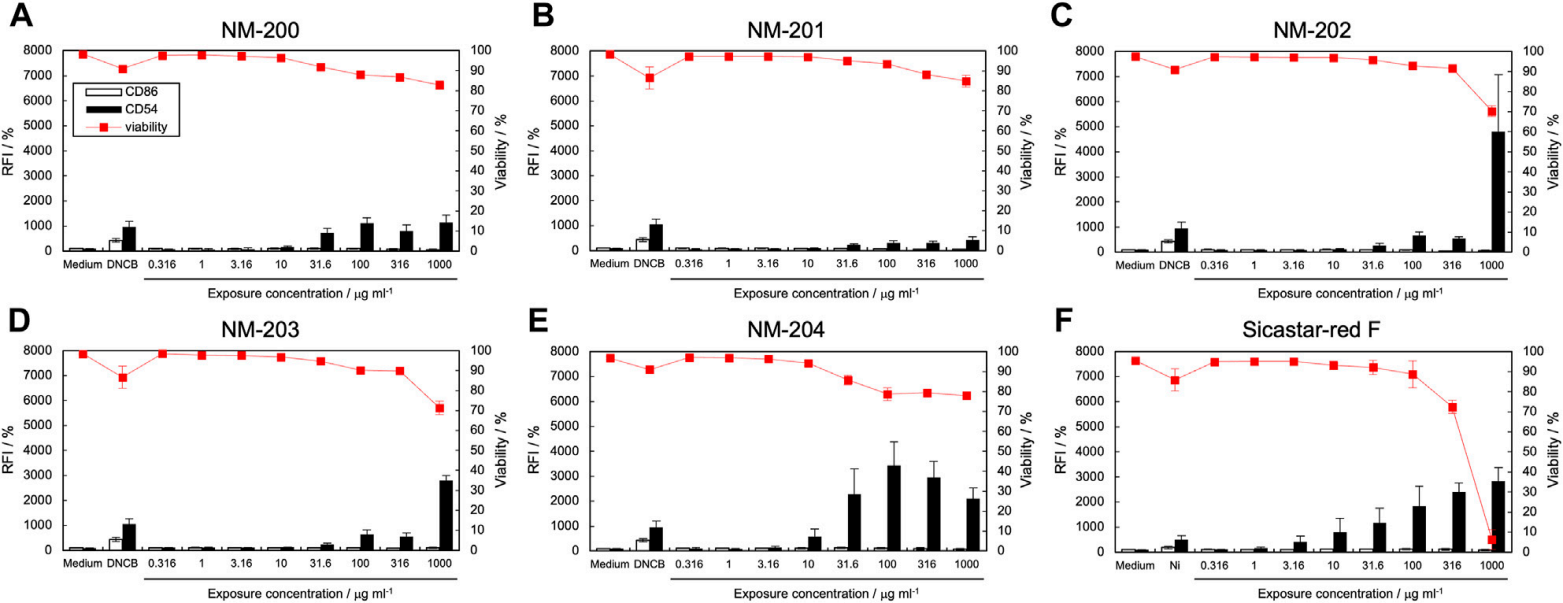
Effect of silica NPs on CD86 and CD54 expression. The expression of CD86 and CD54 and viability of THP-1 cells after exposure to silica NPs [NM-200 **(A)**, NM-201 **(B)**, NM-202 **(C)**, NM-203 **(D)**, NM-204 **(E)**, and Sicastar-red F **(F)**] were quantified using flow cytometry. Viability for Sicastar-red F may be affected by the fluorescent modification of the particles. Each experiment was performed in triplicate, and the results are shown as mean ± standard deviation (S.D.); RFI, relative fluorescent intensity.

Figure 2 shows the expression levels of CD86 and CD54 and cell viability after exposure to silica NP dispersions. Table 2 lists the calculated EC150, EC200, and CV75 values. When exposed to five types of silica NPs (NM-200, NM-201, NM-202, NM-203, and NM-204) synthesized by precipitation and thermal methods, CD54 expression increased significantly; however, the expression levels of CD86 were not elevated (Figures 2A–E). On the contrary, when exposed to Sicastar-red F synthesized by the Stöber process, the expression levels of both CD54 and CD86 increased (Figure 2F). [Figure 2 near here] In addition, the maximum expression level of CD54 after exposure to NM-202 and NM-203 synthesized by the thermal method was higher than that observed in response to NM-200, NM-201, and NM-204 synthesized by the precipitation method. The difference in the activation of THP-1 cells, depending on the type of silica NPs, can be explained based on dispersibility derived from synthesis methods and hydrodynamic diameters. The Stöber method involves alkoxysilane, a silica source, which is hydrolyzed and polycondensed in a mixed solvent of ammonia, water, and ethanol to obtain uniform and monodisperse particles (Stober et al., 1968). As shown in Supplementary Table S1, the dispersibility of Sicastar-red F synthesized by the Stöber method was the highest, while it was higher for NM-202 and NM-203 synthesized by the thermal method than that for NM-200, NM-201, and NM-204 synthesized by the precipitation method. This suggests that dispersibility derived from the synthesis method significantly affects the ability of silica NPs to activate APCs. Furthermore, comparing the particles synthesized by the precipitation method based on the order of maximum CD54 expression (NM-204 > NM-200 > NM-201) suggests that the smaller-sized particles exhibit a higher ability to activate APCs. Unlike silver NPs, ions were not eluted from the silica NPs, suggesting that the degree of THP-1 cell activation was mainly affected by the cellular uptake of silica NPs based on their size. However, the physicochemical properties of silica NPs are not only size-dependent but also vary based on other factors, such as surface modification and porosity. In addition, silica NPs can induce inflammasome activation in macrophages through recognition by the class B scavenger receptor SR-B1 (Tsugita et al., 2017). Therefore, the affinity towards SR-B1 can affect the cellular uptake of silica NPs. Recently, another scavenger receptor, SR-A1, is identified as receptor of silica NPs (Nishijima et al., 2017b). Interestingly, inflammatory response by silica NP through this receptor showed size specific. In a recent report, protein corona formation on the surface of silica particles showed sensitization potential to THP-1 cells (Eto et al., 2022). Hence, it is a forthcoming challenge to increase the variety of test materials and analyze the relationship between APCs activation and other properties, including affinity to SR-B1 and protein absorption properties.

### 3.4 Effect of TiO_2_ NPs on CD86 and CD54 expression by THP-1 cells

**FIGURE 3:**
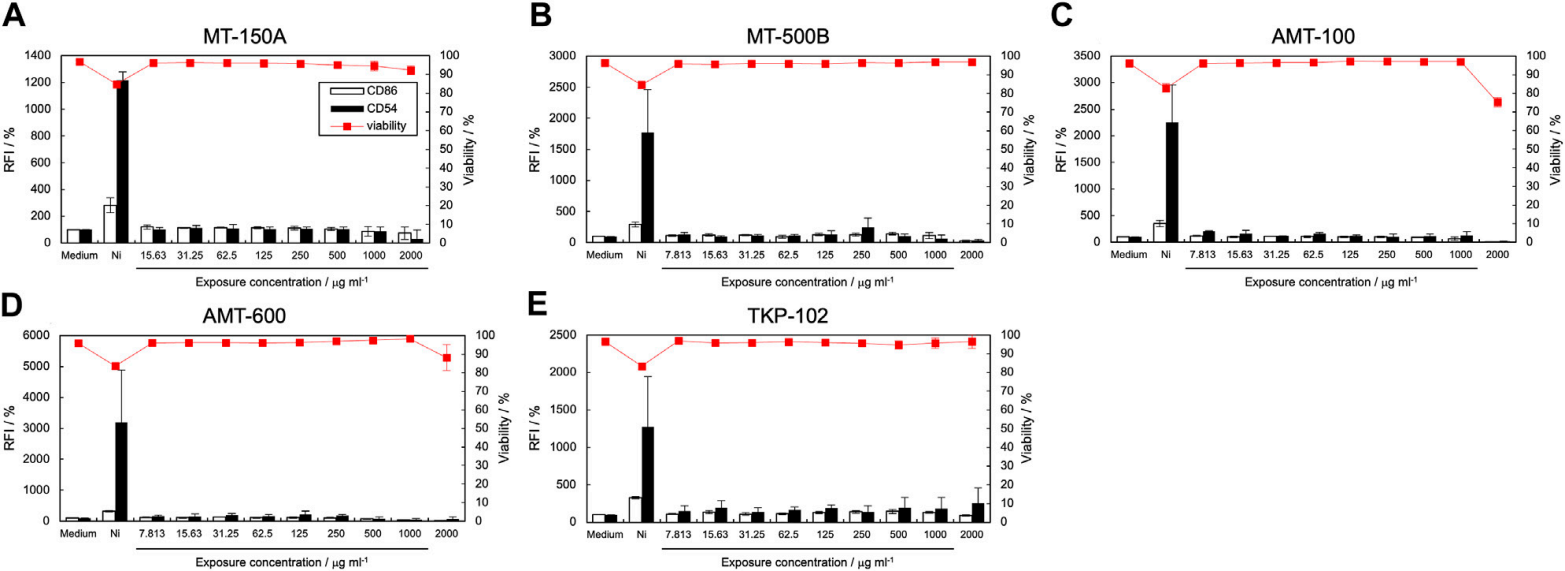
Effect of titanium dioxide NPs on CD86 and CD54 expression. The expression of CD86 and CD54 and viability of THP-1 cells after exposure to titanium dioxide nanomaterials [MT-150A **(A)**, MT-500B **(B)**, AMT-100 **(C)**, AMT-600 **(D)**, and TKP-102 **(E)**] were quantified using flow cytometry. Each experiment was performed in triplicate, and the results are shown as mean ± standard deviation (S.D.); RFI, relative fluorescent intensity.

The expression levels of CD86 and CD54 and cell viability post-exposure to TiO_2_ NP dispersions are shown in Figure 3, and the calculated EC150, EC200, and CV75 values are listed in Table 2. The expression of CD86 and CD54 did not increase when exposed to MT-150A and MT-500B (Figures 3A, B). However, exposure to AMT-100 and AMT-600 increased the expression levels of CD54 (Figures 3C, D). In addition, the expression levels of CD86 and CD54 increased after exposure to TKP-102 (Figure 3E). [Figure 3 near here] On comparing the TiO_2_ NPs, the expression of CD54 exceeded the criteria in only the anatase-type NPs (AMT-100, AMT-600, and TKP-102) but not in the rutile-type particles (MT-150A and MT-500B). Anatase-type NPs have been reported to be more toxic than rutile-type NPs due to reactive oxygen species (ROS) production and DNA damage; moreover, the amount of intracellular uptake and ion release from the NPs is extensive (De Matteis et al., 2017; Minghui et al., 2023). Furthermore, it was revealed that anatase-type TiO_2_ NPs had a more substantial effect on enhancing the immune response to the antigen than rutile-type TiO_2_ NPs due to higher production of cytokines such as IL-1β, IL-10, interferon (INF)-α, IgE, and IgG (Vandebriel et al., 2018). These results are consistent with previous reports, suggesting that h-CLAT could correctly evaluate the difference in immunotoxicity potential based on the TiO_2_ crystal type. Among the anatase-type TiO_2_ NPs, the expression of CD86 exceeded the criteria in TKP102 and but not in AMT-100 and AMT-600. DLS measurements revealed that the hydrodynamic diameter (secondary particle size) of TKP-102 was smaller than other particles of the same rutile-type TiO_2_ NPs. The mechanism of uptake of NPs into cells depends on the secondary particle size, and NPs with a diameter of 30–50 nm are most efficiently taken up; the larger the size, the more difficult it is to take up (Arnida and Ghandehari, 2010). Therefore, the secondary particle size, not the primary particle size, is considered to affect the APC activation ability, and the smaller-sized particles have a higher tendency to activate APCs.

### 3.5 h-CLAT-based evaluation of the immunotoxicity potential of NMs

Our results indicate that the expression levels of CD86 and CD54 in THP-1 cells, based on the h-CLAT procedure, could be used to evaluate the immunotoxicity potential of NMs. h-CLAT is an internationally standardized method for assessing the skin sensitization potential of chemicals with guaranteed reliability (OECD, 2018). Therefore, the h-CLAT-based method is useful in evaluating and comparing the activation potentials of APCs using various types of NMs *in vitro*. Examination of different kinds of TiO_2_ NPs and silica NPs with varied physicochemical properties is essential for a detailed analysis of the relationship between the physicochemical properties and activation of APCs. Additionally, other types of NMs, such as carbon nanotubes, need to be investigated in the future. In order to establish the methods to evaluate the ability of NPs to activate APC *in vitro*, it is also very important to develop standardized methodologies for preparation of NP suspension and applying them to cells. In addition, proinflammatory cytokines such as TNF-α, IL-8, IL-1β, and IL-6 are considered useful indicators of APC activation. In our preliminary experiments, exposure to silica NPs increased the expression of *IL-1B* and *MMP-12* genes and tend to increase the expression of *CCL-3* genes in THP-1 cells (Supplementary Figure S1). Therefore, the expression of such genes can be indicators of activation of APC by NMs. Each type of NMs has different physical properties and exhibits unique characteristics based on their physical properties, therefore, it is necessary to evaluate various NMs and confirm reproducibility to utilize these indicators.

Another important point is how the APC activation by NMs evaluated *in vitro* is linked to their *in vivo* toxicity including from an aspect of exposure level. To establish an AOP for NM toxicity, it is necessary to investigate the causal relationship between cell activation and tissue injury (Halappanavar et al., 2021). For these purposes, the mechanism of cellular activation by NMs, such as cellular uptake through membrane receptors and activation of NF-kB and inflammasome (Nakayama, 2018), should be clarified at the cellular and molecular levels, and *in vitro* assay systems based on h-CLAT would help in elucidating such mechanisms. It is very important to construct a consistent and reliable *in vitro* to *in vivo* extrapolation (IVIVE) model.

## 4 Conclusion

In this study, we evaluated the immunotoxicity potential of various types of NMs based on the expression of CD86 and CD54 in THP-1 cells after exposure to NMs. The ability to activate APCs differs depending on the chemical composition, synthesis method, hydrodynamic diameter, and crystal type of the NMs, suggesting the importance of these factors in APC activation. The APC activation ability of NMs evaluated using THP-1 cells is expected to be a valuable method for assessing the immunotoxicity potential of NMs and elucidating their mechanisms of immunotoxicity.

## Data Availability

The original contributions presented in the study are included in the article/Supplementary Material, further inquiries can be directed to the corresponding author.
