# Supplementary material for: Evaluation of the immunotoxicity potential of nanomaterials using THP-1 cells
Source: Front Toxicol. 2024 Jul 1;6:1293147. doi: 10.3389/ftox.2024.1293147 (PMC11247007; doi:10.3389/ftox.2024.1293147)
Supplement: Supplementary file 1 [file Table1.docx]

Supplementary Material

# Supplementary Figure

Sicastar-red F were exposed to THP-1 in the same procedure as in 2.3. Total RNA was extracted using the RNeasy mini kit (QIAGEN, Hilden, Germany) and cDNA was synthesized using ReverTra Ace^®^ qPCR RT Master Mix with gDNA Remover (TOYOBO, Osaka, Japan). The expression of *IL-1B*, *MMP-12*, *CCL-3* and *GAPDH* genes were evaluated using real-time PCR with THUNDERBIRD^®^ SYBR^TM^ qPCR Mix (TOYOBO). The expression of each gene was quantified by the ΔΔCT method with *GAPDH* gene as an endogenous control and relative expression to control samples were shown. The primers for real-time PCR are 5'-ACAGATGAAGTGCTCCTTCCA-3' (forward) and 5'-GTCGGAGATTCGTAGCTGGAT-3' (reverse) for *IL-1B* (1), 5’-TGCTGATGACATACGTGGCA-3' (forward) and 5’-AGGATTTGGCAAGCGTTGG-3' (reverse) for *MMP-12* (2), 5'- TGCAACCAGTTCTCTGCATC-3' (forward) and 5’- TTTCTGGACCCACTCCTCAC-3' (reverse) for *CCL-3* (3)*,* and 5’- AGCAAGAGCACAAGAGGAAGAGA-3’ (forward) and 5’- GAGGGGAGATTCAGTGTGGTG-3' (reverse) for *GAPDH* (4), respectively.


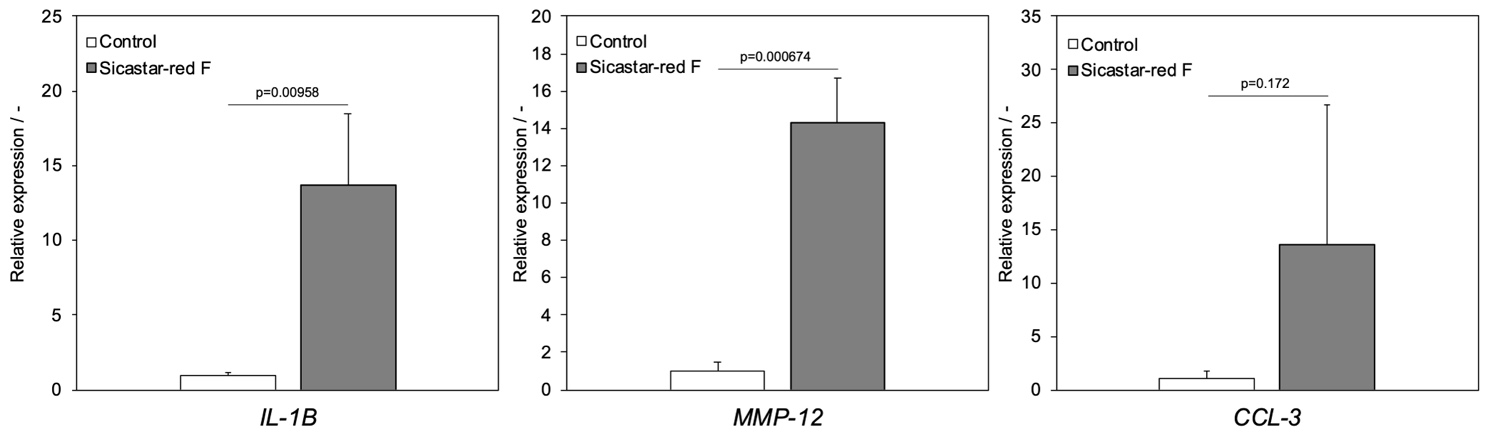


**Figure S1** The expression of *IL-1B*, *MMP-12* and *CCL-3* genes in THP-1 cells after exposure to Sicastar-red F and the results are shown as mean ± standard deviation (S.D.).

# Supplementary Table

**Table S1** Dispersion states of the stock and working solutions

| Name | Solvents | Dispersion state | | | |
| --- | --- | --- | --- | --- | --- |
|  |  | Stock solution (20 mg/ml) | | Working solution (1 mg/ml) | |
|  |  | Immediately  after preparation | 1 hour  after preparation | Immediately  after preparation | 1 hour  after preparation |
| Sicastar-red F | Ultrapure water | Well dispersed | Well dispersed | Well dispersed | Well dispersed |
|  | PBS | Well dispersed | Well dispersed | Well dispersed | Well dispersed |
| NM-200 | Ultrapure water | Well dispersed | Well dispersed | Well dispersed | Precipitated |
|  | PBS | Precipitated immediately | Precipitated | Well dispersed | Precipitated |
| NM-201 | Ultrapure water | Well dispersed | Well dispersed | Well dispersed | Precipitated |
|  | PBS | Well dispersed | Precipitated | Well dispersed | Precipitated |
| NM-202 | Ultrapure water | Well dispersed | Well dispersed | Well dispersed | Precipitated |
|  | PBS | Well dispersed | Well dispersed | Well dispersed | Precipitated |
| NM-203 | Ultrapure water | Well dispersed | Well dispersed | Well dispersed | Precipitated |
|  | PBS | Well dispersed | Well dispersed | Well dispersed | Precipitated |
| NM-204 | Ultrapure water | Well dispersed | Precipitated | Well dispersed | Precipitated |
|  | PBS | Well dispersed | Precipitated | Well dispersed | Precipitated |

**References**

1. Miura K, Matsuo J, Rahman MA, Kumagai Y, Li X, Rikihisa Y. Ehrlichia chaffeensis induces monocyte inflammatory responses through MyD88, ERK, and NF-κB but not through TRIF, interleukin-1 receptor 1 (IL-1R1)/IL-18R1, or toll-like receptors. Infection and Immunity (2011) 79:4947–56.

2. Xie S, Issa R, Sukkar MB, Oltmanns U, Bhavsar PK, Papi A, Caramori G, Adcock I, Chung KF. Induction and regulation of matrix metalloproteinase-12 in human airway smooth muscle cells. Respiratory Research (2005) 6:148.

3. Marischen L, Englert A, Schmitt AL, Einsele H, Loeffler J. Human NK cells adapt their immune response towards increasing multiplicities of infection of Aspergillus fumigatus. BMC Immunology (2018) 19:39.

4. Iijima K, Ishikawa S, Sasaki K, Hashizume M, Kawabe M, Otsuka H. Osteogenic differentiation of bone marrow-derived mesenchymal stem cells in electrospun silica nonwoven fabrics. ACS Omega (2018) 3:10180–7.
